# Low-dose temozolomide before dendritic-cell vaccination reduces (specifically) CD4^+^CD25^++^Foxp3^+^ regulatory T-cells in advanced melanoma patients

**DOI:** 10.1186/1479-5876-11-135

**Published:** 2013-05-31

**Authors:** Laura Ridolfi, Massimiliano Petrini, Anna Maria Granato, Giusy Gentilcore, Ester Simeone, Paolo Antonio Ascierto, Elena Pancisi, Valentina Ancarani, Laura Fiammenghi, Massimo Guidoboni, Francesco de Rosa, Linda Valmorri, Emanuela Scarpi, Stefania Vittoria Luisa Nicoletti, Stefano Baravelli, Angela Riccobon, Ruggero Ridolfi

**Affiliations:** 1 Immunotherapy Unit, Istituto Scientifico Romagnolo per lo Studio e la Cura dei Tumori (IRST), IRCCS, Meldola, FC, Italy; 2 Melanoma, Cancer Immunotherapy and Innovative Therapies Unit, Istituto Nazionale per lo Studio e la Cura dei Tumori, Fondazione “G. Pascale”, Naples, Italy; 3 Unit of Biostatistics and Clinical Trials, IRST IRCCS, Meldola, Italy; 4 Blood Transfusion Unit, Morgagni Pierantoni Hospital, Forlì, Italy

**Keywords:** Vaccine, Melanoma, Dendritic cell, Foxp3+Tregs, Low-dose temozolomide

## Abstract

**Background:**

In cancer immunotherapy, dendritic cells (DCs) play a fundamental role in the dialog between innate and adaptive immune response, but several immunosuppressive mechanisms remain to be overcome. For example, a high number of CD4^+^CD25^++^Foxp3^+^ regulatory T-cells (Foxp3+Tregs) have been observed in the peripheral blood and tumor microenvironment of cancer patients. On the basis of this, we conducted a study on DC-based vaccination in advanced melanoma, adding low-dose temozolomide to obtain lymphodepletion.

**Methods:**

Twenty-one patients were entered onto our vaccination protocol using autologous DCs pulsed with autologous tumor lysate and keyhole limpet hemocyanin. Patients received low-dose temozolomide before vaccination and 5 days of low-dose interleukin-2 (IL-2) after vaccination. Circulating Foxp3+Tregs were evaluated before and after temozolomide, and after IL-2.

**Results:**

Among the 17 evaluable patients we observed 1 partial response (PR), 6 stable disease (SD) and 10 progressive disease (PD). The disease control rate (PR+SD = DCR) was 41% and median overall survival was 10 months. Temozolomide reduced circulating Foxp3+Treg cells in all patients. A statistically significant reduction of 60% was observed in Foxp3+Tregs after the first cycle, whereas the absolute lymphocyte count decreased by only 14%. Conversely, IL-2 increased Foxp3+Treg cell count by 75.4%. Of note the effect of this cytokine, albeit not statistically significant, on the DCR subgroup led to a further 33.8% reduction in Foxp3+Treg cells.

**Conclusions:**

Our results suggest that the combined immunological therapy, at least as far as the DCR subgroup is concerned, effectively reduced the number of Foxp3+Treg cells, which exerted a blunting effect on the growth-stimulating effect of IL-2. However, this regimen, with its current modality, would not seem to be capable of improving clinical outcome.

## Background

Dendritic cells (DCs) present antigens to naïve T-lymphocytes and regulate the activation of the adaptive response, playing a fundamental role in the dialog between innate and adaptive immune response [1]. DCs are suppressed inside tumor tissue.

The tumor microenvironment is immunosuppressive because of the presence of weak self or self-like antigens, the absence of a real danger signal and the presence of tumor immunosuppressive signals. However, recent experiences of DC vaccination in melanoma show that proven immunostimulation usually correlates with positive clinical outcome [2].

A review of 54 DC vaccination trials showed that the use of mature DCs (mDCs) rather than immature DCs (iDCs), the adjuvants of choice and confirmed immunization strongly influence clinical outcome [3]. The authors of the review concluded that DC vaccination must aim to enhance antigen-specific cytotoxic T-cells and to decrease immunosuppression, perhaps through CD4+CD25++Foxp3+ regulatory T cell (Foxp3+Treg) lymphodepletion or through the use of new molecules, for example, anti-CTLA-4 or anti-PD1, or immunostimulators, e.g. CpG [4-7]. Foxp3+Tregs, a subset of CD4+ T cells, were first described by Sakaguchi [8], who discovered that these cells constitutively express high levels of interleukin-2 (IL-2) receptor α chain (CD25), preventing autoimmune diseases in mice. These cells have since been shown to be involved in the development of autoimmunity, allergy, and rejection in transplant medicine and in the suppression of immune responses to cancer.

Higher numbers of Foxp3+Tregs have been observed in the peripheral blood of cancer patients, in ascites, in the tumor microenvironment, and in tumor draining lymph nodes in a variety of solid cancers [9]. There appears to be a two-way relationship between DCs and Foxp3+Tregs. iDCs are capable of inducing the peripheral formation of Foxp3+Tregs [10]. Furthermore, mDCs are able to convert resting autologous T-cells into Foxp3+Tregs via an indoleamine 2,3-dioxygenase mechanism. This method of Foxp3+Treg expansion is still dependent on CD80/CD86 ligation and endogenous IL-2 production [11]. Foxp3+Treg depletion can evoke effective tumor immunity and has led to tumor rejection in several animal models [8,12]. Although it is not clear whether chemotherapy can be used to counter Foxp3+Treg-derived tumor protection, it would, however, seem to activate the immunocompetence needed for vaccine response [13]. Low-dose metronomic cyclophosphamide regimens have been shown to deplete the Foxp3+Treg cell population in humans [14]. However, it remains uncertain whether other chemotherapeutic agents can induce such Foxp3+Treg depletion. Temozolomide (TMZ), for example, is capable of modulating blood Foxp3+Tregs. In phase III trials TMZ and dacarbazine have shown similar response rates [15,16]. TMZ has also been used in association with low-dose IL-2 in patients with advanced melanoma, producing durable clinical responses [17], and in combination with a telomerase peptide vaccination, showing encouraging results [18]. In 2009 Banissi and coworkers reported that low-dose metronomic TMZ regimens (0.5 and 2 mg/kg for 21 days) induced a significant decrease in Foxp3+Treg/CD4+ ratios in the spleen of tumor-bearing animals [19]. Similarly, Su and coworkers observed a specific decrease in CD4+ T-cells in patients treated with TMZ [20].

On the basis of these observations we decided to make an amendment of our study [21] protocol on dendritic cell-based vaccination in advanced melanoma by adding low-dose TMZ in an attempt to deplete the Foxp3+Treg population. The present study presents the results obtained from this amendment.

## Methods

### Patients

From January 2007 to January 2009, 21 patients with advanced melanoma were entered onto a vaccination protocol using autologous DCs pulsed with autologous tumor lysate (ATL) or autologous tumor homogenate (ATH) and keyhole limpet hemocyanin (KLH). Low-dose TMZ was administered before vaccination, as per the study amendment. Inclusion criteria were age < 70 years, histologically confirmed diagnosis of melanoma, measurable disease (excluding brain metastases), previous removal of one or more metastatic lesions from which a sufficient quantity of ATL/ATH had been obtained for at least 6 vaccinations, performance status (PS) ≤ 2 (according to ECOG criteria), life expectancy > 4 months.

### Treatment

The first 16 patients were treated (only for the first 6 cycles) in the 14 days before vaccination with low-dose TMZ: 75 mg/m^2^/day (max 100 mg/day) (days −14 to −1). In an attempt to further improve compliance to treatment, the last 5 patients received the same dosage/day of TMZ for only 7 days before each vaccination (day −7 to −1).

**Figure 1:**
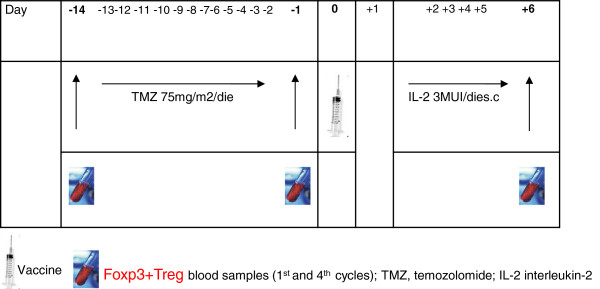
**Flow-chart: treatment and Foxp3+Treg blood sample schedule.**

Patients were then vaccinated with mDCs pulsed with ATL or ATH via intradermal injection using an insulin needle. Several injections were made (average 6–8) near the groin or the armpit in non metastatic, non lymphadenectomized sites every two weeks for the first 4 vaccinations and then monthly thereafter until progression occurred or for a maximum of one year. Subcutaneous IL-2 (Novartis, Italy) 3,000,000 IU/day was administered 48 hrs after each vaccination for 5 consecutive days (from days +2 to +6) (Figure 1). Delayed-type hypersensitivity (DTH) assessment was carried out before the first vaccination, after the 4th vaccination, and every 2 vaccinations thereafter. The disappearance of or an important reduction in pre-existing lesions in concomitance with disease progression in other sites was considered as a mixed response (MR). The best response obtained was considered for evaluation purposes. Toxicity or adverse events were assessed after each vaccine administration. Circulating Foxp3+Tregs were evaluated on days −14, 0 and immediately after IL-2 during the first and the fourth cycle (Figure 1).

The study protocol and, subsequently, the amendment, were reviewed and approved by the local Ethics Committee (2001 and 2007, respectively), in accordance with ethical standards laid down in the 1964 Declaration of Helsinki, and authorized in July 2001 by the Italian Ministry of Health. All patients gave informed written consent to receive treatment.

### Autologous tumor lysate (ATL) preparation

Surgically removed tumor samples were mechanically dispersed to create a single-cell suspension. The largest pieces were incubated at 37°C in enzyme mix (collagenase 0.1%, hyaluronidase 0.01%, DNAse 0.1%, Sigma, Milan, Italy) in RPMI 1640, (PAA Laboratories GmbH, Pasching, Austria) for 3 h. At the end of incubation the pellets were washed 3 times with PBS and incubated for at least 20 min in sterile distilled water. Lysis was monitored by light microscope. Larger particles were removed by centrifugation (10 min at 600 *g*) and the supernatant was passed through a 0.2-μm filter. Protein contents were determined and aliquots, after verifying sterility, were stored at −80°C until use.

### Autologous tumor homogenate (ATH) preparation

In some cases the surgically removed tissue was stored at −80°C because the decision to vaccinate had still not been taken. Frozen tissue fragments were pulverized in a dismembrator after immersion in liquid nitrogen. Pulverized tissue was then suspended in PBS. After centrifugation, the supernatant was treated as described above.

### DC generation

DC were prepared from peripheral blood monocytes (PBMCs) obtained by leukapheresis without previous mobilization. Five to nine liters of blood were processed in each collection. PBMCs were purified on Ficoll-Paque. An aliquot of PBMCs was utilized immediately for DC generation and the rest was frozen in bags and cryopreserved in nitrogen vapors for use at a later date (4–5 bags/collection). Fresh PBMCs or thawed PBMCs were incubated in tissue culture flasks with CellGro DC medium (Cell Genix, Freiburg, Germany) at 10 × 10^6^ cells/ml for 2 h. Non-adherent cells were discarded and adherent cells were incubated in CellGro DC medium containing 1000 IU/ml rhIL-4 (Cell Genix, Freiburg, Germany) and 1000 IU/ml rhGM-CSF (Cell Genix) for 7 days to generate a DC-enriched cell population. On day 6, 90% of the DC culture was pulsed with ATL/ATH (100 mg/ml), while the remaining 10% was pulsed with KLH (50 mg/ml). Both cultures were then incubated overnight. On day 7, the cells were defined as immature DCs (iDCs). After eliminating the previous culture medium, pulsed iDCs were cultured for a further 2 days with a cocktail of cytokines (TNFα, IL-1β, IL-6, Cell Genix; PGE2, Pfizer, Latina, Italy). On day 9 they were defined as mature DCs (mDCs). mDCs (median 10×10^6^, range 4.9–17 × 10^6^) were removed, washed, suspended in sterile saline solution and sent immediately for therapeutic injection. The vaccine was maintained at room temperature and administered within one hour of preparation. The entire process, from leukapheresis to injection, was carried out in accordance with Good Manufacturing Practice (GMP) guidelines.

### Quality control of dendritic cell vaccine

#### Safety test

Endotoxin (≤ 0.5 EU/ml), germ-free and mycoplasma-free tests were performed in accordance with European Pharmacopoeia guidelines.

#### Phenotypic analysis

DC vaccine phenotype was determined by flow cytometry using a BD FACSCanto flow cytometer (Becton Dickinson, Milan). The following monoclonal antibodies with related isotype controls were used: anti-CD80, anti-CD86, anti-HLA-DR (BD Pharmingen, San Diego, CA, USA), and anti-CD83 (Beckman Coulter, Milan, Italy).

### Analysis of CD4+CD25++Foxp3+ regulatory T cells

Foxp3+Treg levels (%) in whole blood CD4^+^ lymphocytes were measured using a flow cytometry assay. Cells were stained with combinations of the following antibodies: Foxp3 Alexa Fluor 488/CD4 PE-Cy5/CD25 PE (Biolegend; San Diego, CA, USA). Test tubes were labeled, incubated in the dark for 30 min and then washed with phosphate buffered saline. For intracellular staining of Foxp3, samples were fixed and permeabilized using Foxp3 Fix/Perm and Perm Buffer (Biolegend) according to the manufacturer’s instructions.

Data acquisition and analysis were performed using the FACS ARIA II flow cytometry system and FACS Diva™ software (BD Biosciences; Mountain View, CA, USA). Lymphocytes were gated via their forward and side scatter properties, and T-cells were identified on the basis of their expression of CD4, CD25 high and Foxp3. The absolute concentration of Foxp3+Treg cells was quantified by determining the percentage of fluorescence-positive cells within the forward/side scatter lymphocyte gate, and by multiplying this percentage by the absolute lymphocyte concentration determined using an automated hematology analyzer, Sysmex XE2100, (Sysmex Europe GmbH, Hamburg-Norderstedt, Germany). As this furnished a phenotypic evaluation of Foxp3+Treg levels, no functional or suppression assays were performed to assess Foxp3 + Treg levels.

### *In vivo* monitoring (DTH)

ATL or ATH (10 μg) and KLH (5 μg) were each suspended in 500 μl of PBS and injected intradermally into the forearm of the patient. PBS alone was used as negative control.

### Statistical evaluations

Survival was calculated as the time between the date of the first treatment cycle and the date of death from any cause. Non-parametric ranking statistics (median test) were used to analyze the relationship between the percent change and patient status (progression or clinical benefit). The paired *T*-test was used to determine the level of significance in lymphocyte count changes from baseline to T1 or T2. Statistical analyses were carried out with SAS Statistical software (version 9.3, SAS Institute, Cary, NC, USA).

## Results

### Characteristics of DCs

DC vaccine was generated successfully from all the patients. Leukapheresis provided a sufficient number of PBMCs (median, 3.5×10^9^; range 1.7–9×10^9^) to prepare an average of 5 cycles of vaccine (range 3–8 cycles) per patient. All vaccines were found to be compliant with the safety testing. The analysis of mDC lineage markers, carried out on dendritic cells obtained from both fresh and frozen PBMCs, revealed high expression of CD83, HLA-DR, CD80 and CD86.

### Clinical results

Patients were required to undergo a minimum of 4 vaccinations to be considered evaluable, and 17 out of 21 patients satisfied the criteria. Of the 4 patients who were not assessable, one experienced severe disseminated intravascular coagulation before the first cycle, 2 showed a rapid worsening of clinical conditions and did not complete the first treatment cycle and one developed symptomatic brain metastases after the first vaccination. Patient characteristics were as follows: 12 males, 5 females, median age 57 years (range 35–73), 4 stage M1a, 3 M1b and 10 M1c, all pretreated (Table 1). The first 12 evaluable patients received a median TMZ dose/cycle of 1400 mg, while the remaining 5 were administered 700 mg. Only 2 patients were forced to reduce the dose of TMZ because of intolerance (Table 2). No major toxicities were observed. The most frequent adverse events to TMZ were grade II nausea and vomiting. There were no cases of hematological toxicity. Toxicities linked to vaccination plus IL-2 were mainly flu-like syndromes (grade I-II asthenia and fever) after IL-2 administration and local reactions in the vaccine injection sites.

**Table 1:** Patient characteristics

**Patient ID**	**Gender**	**Age (years)**	**Site of evaluable disease**	***M1 *classification**	**Previous treatments**
28	M	66	Lymph nodes, lung, liver, pelvis	a	BIOCT
29	F	43	Lymph nodes, lung, liver, skin	c	BIOCT
30	M	69	Lymph nodes, skin, bone	c	BIOCT
31	M	57	Lymph nodes, skin	a	BIOCT
32	M	59	Lymph nodes, lung, liver	c	BIOCT
33	F	44	Liver, skin	c	BIOCT, anti-CTLA4Ab (interrupted after 2 cylces due to grade IV toxicity)
34	F	45	Lung, lymph nodes, soft tissue	b	CT and RT
35.	M	73	Lymph nodes, soft tissue, peritoneum	a	Surgery and RT
36	M	68	Skin, lung	b	BIOCT
37	F	35	Pelvis, lymph nodes,skin, peritoneum, lung	b	CT, anti-CTLA4Ab
38	M	51	Lymph nodes, skin, lung	c	anti-CTLA4Ab, CT
39	M	62	Lymph nodes, skin, adrenal gland	c	Leg Stopflow CT, ECT
40	M	47	Lymph nodes,skin, peritoneum	c	anti-CTLA4Ab, CT, RT
41	F	57	Lymph nodes	a	IFN, antiCD137Ab, CT
42	M	49	Lymph nodes, soft tissue, colon	c	CT
43	M	60	Adrenal gland, soft tissue, lung	c	CT
44	M	57	Lung, liver, lymph nodes, soft tissue	c	CT, IFN
**Summary**	**Male** 12	**Median**		**M1a**:**4**	
				**M1b**:**3**	
	**Female** 5	**57** (range 35–73)		**M1c**:**10**	

*M* Male, *F* Female, *BIOCT* Biochemotherapy, *CT* Chemotherapy, *RT* Radiotherapy, *INF* Interferon-alfa, *ECT* Electrochemotherapy, *anti*-*CTLA4Ab*, antibody against CTLA4, *antiCD137Ab*, antibody against.

**Table 2:** **Median dose of temozolamide**, ***in vivo *immunologial and clinical response**, **duration of response and median overall survival**

**Patient ID**	**Median dose/cycle of induction temozolamide (mg) first 4 cycles**	**No. vaccinations**	**Median no. of cells administered 10**^**6 **^**(range)**	**DTH Best response after 4 or more vaccinations L/H KLH**		**Vitiligo**	**Clinical response**	**Response duration (months)**	**OS (months)**	**After vaccination treatments**
**28**	1400	12	12.6	**+++**	**+++**	-	**SD**	9	**12**	None
			(11.5–17)							
**29**	1400 first 2 cycles, 700 last 2 cycles	4	7.7	**–**	**–**	–	PD		3	None
			(5.4–8.6)							
**30**	1400	5	11,9	**–**	**–**	–	PD		6	None
			(11.5–14)							
**31**	1400	8	12.6	**–**	**–**	–	**SD**	4	**7**	None
			(11.1–15.3)							
**32**	1400 first 3 cycles, 700 last cycle	5	8.5	**–**	**–**	–	PD		6	None
			(7.5–10)							
**33**	1400	8	8.8	**+++**	**+++**	+	**SD**	4	**12**	BIOCT
			(4.9–10)							
**34**	1400	6	10.1	**–**	**–**	–	PD		14	None
			(6.9–14.5)							
**35**	1400	17	9.4	**++**	**–**	–	**SD**	10	**36+**	antiCTLA4Ab
			(6.5–10.8)							
**36**	1400	6	9.8	**+**	**+**	–	**SD**	9	**41+**	antiCTLA4Ab
			(8.3–13.9)							
**37**	1400 first cycle, 700 last 3 cycles	17	7.8	**++**	**–**	–	**SD**	4	**16**	None
			(5.7–10)							
**38**	1400	11	9.3	**–**	**+**	–	PD		12	None
			(8.1–10.8)							
**39**	1400	5	10	**–**	**–**	–	PD		7	None
			(10–10)							
**40**	1400	5	11.3	**–**	**–**	–	PD		4	None
			(10–15)							
**41**	700	10	9.3	**–**	**+++**	–	**PR**	6	**14**	None
			(5.8–10)							
**42**	700	4	10	**–**	**–**	–	PD		4	None
			(10–10)							
**43**	700	5	10	**+**	**+**	–	PD		8	None
			(9.6–13.8)							
**44**	700	7	10	**–**	**+**	–	PD		10	antiCTLA4Ab
			(10–10)							

*BIOCT* Biochemotherapy, *anti*-*CTLA4Ab* antibody against CTLA4, *PD* Progressive disease, *SD* Stable disease,*PR* Partial response, *Os* Overall survival, *DTH* Delayed-type hypersensitivity, *KLH* Keyhole limpet hemocyanin, *L*/*H* Lysate/homogenate, + Patients alive in December 2011.

Up to December 2011 (last follow up) 1 partial response (PR) of 6 months (one of the 5 patients who received TMZ for 7 days), 6 cases of stable disease (SD) with a median duration of 6.5 months (among whom another patient from the group given 7-day TMZ) and 10 cases of progressive disease (PD) had been observed. The disease control rate (DCR) was 41% (7/17 patients). Median overall survival (OS) was 10 months (95% CI 6–14) with a median follow up of 38 months (range 3–41). Six of the 7 patients who achieved disease control (DCR) had positive DTH: one patient who obtained PR showed positivity for KLH alone, while 5 SD patients were positive to lysate or homogenate. The remaining SD patient had negative DTH tests. Among those who progressed, 3 showed weak positivity to DTH (Tables 2 and 3).

**Table 3:** Clinical response in 17 evaluable patients

	**No. (%)**	**Clinical benefit**	**Median OS (months)**
**PR**	1 (5.8)	7/17 (41%)	14
**SD**	6 (35.2)		
**PD**	10 (58.8)		6.5
**All 17 patients**			10

*PR* Partial response, *SD* Stable disease, *PD* Progressive disease, *OS* Overall survival.

Only 4 patients underwent further treatment after vaccination, 3 of whom with ipilimumab. A further 4 patients were treated before vaccination with ipilimumab. It cannot be ruled out that OS was not influenced by treatment with this monoclonal antibody.

### CD4+CD25++Foxp3+ regulatory T cells

**Figure 2:**
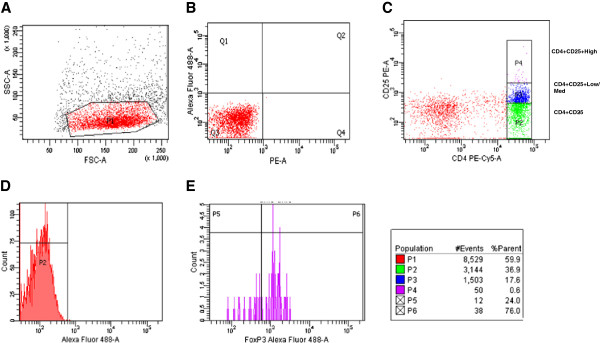
**Flow cytometry detection of CD4+CD25++.** (**A**) A dot plot of forward scatter (FSC) and side scatter (SSC) was used to define the lymphocyte population (P1). (**B**-**C**) The expression of CD4 and CD25 total lymphocytes (P1) was detected and compared with that of the negative control and different gates were drawn to define CD4+CD25^_^ cells (P2), CD4+CD25+^low-medium^ cells (P3) and CD4+CD25++ cells (P4). The percentage of CD4+CD25++FoxP3+ cells in total lymhocytes was determinated. (**D**-**E**) The histograms show the percentage of FoxP3+Treg cells compared with the negative control.

Analysis was based on the absolute number of Foxp3+Tregs, and 14 of the 17 evaluable patients (4 of whom had received the 7-day TMZ treatment) had adequate blood samples. A significant decrease of 60.5% in the absolute number of Foxp3+Tregs was observed in these patients after the first treatment cycle, including those who underwent 7-day TMZ (p = 0.017) (Figure 2). Conversely, evaluation during the 4th cycle highlighted a non significant reduction of 5.5% in Foxp3+Tregs (p = 0.288) (Table 4). A 14% reduction in all lymphocytes was observed in this group (p = 0.020).

**Table 4:** Percentage of variation in Foxp3+Treg and lymphocyte count

	**Foxp3+Tregs (%) before and after TMZ**	**All lymphocytes (%) before and after TMZ**	**Foxp3+Tregs after TMZ and IL-2**	**All lymphocytes (%) after TMZ and IL-2**	**Foxp3+Tregs before and after TMZ**	**Foxp3+Tregs after TMZ and IL-2**	**Basal Foxp3+Tregs (%) after IL-2**	**All basal lymphocytes (%) after IL-2**
	**1st cycle**	**1st cycle**	**1st cycle**	**1st cycle**	**4th cycle**	**4th cycle**	**4th cycle**	**4th cycle**
**All patients**	**−60.5 p = 0.017**	**−14.2 p = 0.020**	+75.4	+15	−5.5	+38.6	−20.3	−4.0
**DCR**	−30.2	−11.9	−33.8	−22.2	−5.3	+53.2	−37.4	−16.4
**PD**	**−76.4 p = 0.032**	−17.1	+388.9	+32.9	−22	+38.6	−20.3	+7.8

*TMZ* Temozolomide, *IL*-*2*, Interleukin-2, *DCR* Disease control patients, *PD* Progressive disease patients.

We further divided the 14 patients into 2 groups comprising those who achieved DCR and those who did not (PD). In DCR patients (6), a non statistically significant reduction of 30.2% (range −71.3% to +2.8%) was observed in Foxp3+Tregs (p = 0.161). Similarly, statistical significance was not reached for the 76.4% (range −84.9% to +72.2%) reduction in Foxp3+Tregs observed in PD patients. A median variation of −11.9% (range −30.0% +2.1%) and −17.1% (range −72% +51.7%) in all lymphocytes was registered in DCR and PD patients, respectively p values were not significant in either group.

**Figure 3:**
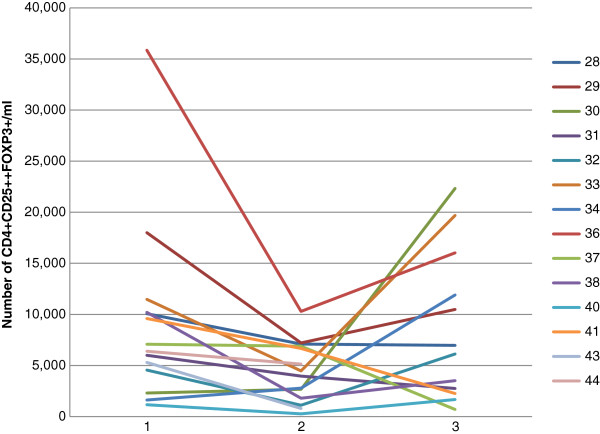
**Foxp3+Treg trend in one patient during the first cycle of temozolomide: basal (1), after temozolomide (2) and after IL-2 (3).**

Taking into consideration the variations from day 0 to after the end of IL-2 treatment, an overall increase of 75.4% was registered in the entire group (only 12 patients were evaluable because post-IL-2 blood samples were not available in 2 DCR patients) during the first cycle (Figure 3). When the 2 groups were analyzed separately, this result converted into a +388.9% increase in PD patients, but a −33.8% reduction in DCR patients. Lymphocyte values were as follows: +15.6% in all 12 patients (range −71.5% +104.5%), +32.9% (range −29.5% +104.5%) in PD and −22.2% (range −71.5% +23.6%) in DCR patients. None of these variations was statistically significant. Interestingly, a non statistically significant increase of +53.2%. was observed for DCR patients during the 4th cycle.

In the 9 patients for whom baseline (time zero) and final (after IL-2 at the end of the 4th treatment cycle) blood samples were available, we observed a −20.3% (range −84.0% to +569.7%) reduction in Foxp3+Tregs, which, however, did not reach statistical significance even when the two groups (DCR and PD) were considered separately. In the same 9 patients we also observed a reduction in the total lymphocyte count of −4.0% (range −76.0% to +32.8%), with values of +7.8% (range:–76.0% to 32.8%) in PD patients and −16.4% (range −33.5% to +16.9%) in DCR patients (median test p = 0.416) (Table 4).

## Discussion

A recent review of results from clinical trials of dendritic cell vaccination shows that immunological response is related to improved clinical outcome [3]. Our previous experience with 27 patients treated with dendritic cell vaccination for metastatic melanoma further confirms this finding in that we observed significantly better overall survival among patients with positive DTH skin testing after treatment (22.9 months positive DTH vs. 4.8 months negative DTH) [22]. The same review by Nakai also underlined the importance of adjuvants aimed at reducing immune suppression (e.g. lowering Foxp3+Treg cell numbers) or at enhancing the immunological stimulation (e.g. anti-CTLA4 or anti-PD1 antibodies). A decrease in Foxp3+Tregs has been reported to improve cytotoxic T cell response and would appear to be related to a better response to vaccines [23-25]. Bjoern and coworkers observed a reduction, albeit not statistically significant, in Foxp3+Treg cell numbers among patients who obtained at least disease stabilization with respect to those who progressed [26]. The authors thus hypothesized that these cells may exert effective immune suppressant action *in vivo* and carried out further research aimed at identifying new strategies to reduce Foxp3+Treg numbers. Metronomic cyclophosphamide, widely employed for this purpose, has achieved conflicting results. Although a recent work reported increased clinical efficacy of DC vaccination when used in combination with this alkylating agent, the number of Foxp3+Tregs remained virtually unchanged [27]. Low-dose IL-2, widely used as an adjuvant after vaccination, has been shown to cause a peak in Foxp3+Tregs after administration which counterbalances the reduction obtained with cyclophosphamide or daclizumab [28,29]. Taking into consideration these data and our own experience, we designed a study in which TMZ was added before vaccination with the aim of reducing the number of circulating Foxp3+Treg cells. The previous schedule comprising adjuvant IL-2 for five days after each dose of vaccine was maintained. Results were not completely satisfactory. Median overall survival was 10 months, somewhat lower than the 16 months reported by our group in a recent update of a previous study [22]. Immunological response, evaluated by the DTH skin test, was also lower. However, in our experience, TMZ reduced circulating Foxp3+Treg cells in all patients, including those who were only treated with the alkylating agent for 7 days. In fact, we observed a statistically significant reduction in Foxp3+Tregs (60%) after the first cycle, whereas the absolute lymphocyte count only decreased by 14%, suggesting that the effect of TMZ is fairly specific for this subpopulation of lymphocytes. Conversely, IL-2 increased the Foxp3+Treg cell count by 75.4% which, albeit not statistically significant, is consistent with published data. Of note, administration of the cytokine in the DCR subgroup led to a further decrease in Foxp3+Treg cells (33.8%), which again was not statistically significant, probably because of the small number of patients involved. This would seem to indicate that combined immunological therapy, *i*.*e*. TMZ plus dendritic cell vaccination plus IL-2, at least for responding patients, effectively reduced the number of Foxp3+Treg cells, which showed a poor or null response to the growth-stimulating effect of IL-2. Of note, PD patients showed a twofold reduction in Foxp3+Tregs after TMZ treatment with respect to DCR patients (−76.4% versus −30.2%, respectively). Furthermore, a +400% increase in Foxp3+Tregs was seen in the PD group after IL-2 administration, whereas values of these lymphocytes continued to diminish (−33.8%) in the DCR group. It can thus be hypothesized that, although PD patients had a higher quota of proliferating lymphocytes that were substantially depleted by chemotherapy, the residual cell quota was still capable of expanding significantly under IL-2 stimulation. Overall, the median Foxp3+Treg cell number dropped by only −20.3% (range −84.0% – +569.7%) between baseline values and the end of IL-2 treatment but was more pronounced in the DCR group (−37.4%; range −56.8% – +13.2%). Responders once again showed a greater reduction, albeit not statistically significant, in Foxp3+Treg cells, without the typical rebound after IL-2. Noteworthy, Foxp3+Treg cell count showed a continuously decreasing trend in this subgroup, and seven days of TMZ seem to have been as effective as 14 days, although this assumption is based on data from only a few patients. In our case series 7 patients also received ipilimumab (4 before and 3 after vaccination), and it cannot be ruled out that this did not influence OS, especially if we take into account the possible impact of the drug on Foxp3+Tregs.

## Conclusions

TMZ reduced the number of circulating Foxp3+Treg cells in all patients, including those who were treated for 7 days only. Of note, administration of the cytokine in the DCR subgroups led to a further decrease in Foxp3+Treg cells (33.8%), which was not, however, statistically significant, probably because of the small number of patients involved. This would seem to indicate that combined immunological therapy, *i*.*e*. TMZ plus dendritic cell vaccination plus IL-2, at least for responding patients, effectively diminshed the number of Foxp3+Treg cells. The effect of treatment on Foxp3+Treg cell levels did not lead to a better clinical outcome, as can be seen from a comparison with historical data. Moreover, 7 patients also received treatment with an anti-CTLA4 antibody, which may have influenced OS and interfered with the clinical course of the disease. Our findings should thus be interpreted with caution. Further studies are needed to evaluate the clinical impact of Foxp3+Treg depletion strategies before vaccine or immunotherapy.

## Abbreviations

DC: Dendritic cells; Foxp3+Tregs: CD4+CD25++Foxp3+ regulatory T cells; IL-2: Interleukin-2; PR: Partial response; SD: Stable disease; PD: Progressive disease; DCR: Disease control rate; mDCs: mature DCs; iDCs: immature DCs; TMZ: Temozolomide; ATL: Autologous tumor lysate; ATH: Autologous tumor homogenate; KLH: Keyhole limpet hemocyanin; PS: Performance status; DTH: Delayed-type hypersensitivity; MR: Mixed response; PBMCs: Peripheral blood monocytes.

## Competing interests

Temozolomide was kindly supplied by Merck Sharp & Dohme Corp. The opinions expressed in this paper are those of the authors and do not necessarily represent those of Merck Sharp & Dohme Corp. The authors have no potential conflicts of interest to declare.

## Authors’ contributions

LR, MP, AMG, RR, EP, VA, SVLN, MG, FDR and AR conceived the idea and designed the study. LR, RR, MG and FDR were responsible for patient enrolment, treatment and follow up. MP, AMG, EP, VA, LF and AR were involved in the preparation of clinical grade lysate/homogenate, dendritic cell vaccine and DTH tests. GG, ES and APA performed Foxp3+Treg staining and analysis. SB carried out leukapheresis for PBMC collection. LR, MP, AMG, RR, ES, LF, EP, VA, MG, FDR, LV and AR were involved in the acquisition, analysis and interpretation of data. LR, MP, AMG, RR and MG drafted the manuscript. All authors read and approved the final manuscript.
